# Determination of the glycosylation-pattern of the middle ear mucosa in guinea pigs

**DOI:** 10.1016/j.ijpharm.2015.02.056

**Published:** 2015-04-30

**Authors:** Elisabeth Engleder, Elisabeth Demmerer, Xueyan Wang, Clemens Honeder, Chengjing Zhu, Christian Studenik, Michael Wirth, Christoph Arnoldner, Franz Gabor

**Affiliations:** a Department of Pharmaceutical Technology and Biopharmaceutics, University of Vienna, Althanstraße 14, 1090 Vienna, Austria; b Department of Otorhinolaryngology, Medical University of Vienna, Währinger Gürtel 18, 1090 Vienna, Austria; c Department of Pharmacology and Toxicology, University of Vienna, Althanstraße 14, A-1090 Vienna, Austria

**Keywords:** OM, otitis media, MEM, middle ear mucosa, WGA, wheat germ agglutinin, STA, *Solanumtuberosum* lectin, UEA-I, *Ulexeuropaeus* isoagglutinin I, LCA, *Lensculinaris* agglutinin, GNA, *Galanthusnivalis* agglutinin, MFI, mean fluorescence intensity, Otitis media, Middle ear mucosa, Guinea pigs, Bioadhesive drug delivery, Lectins

## Abstract

In the present study the glycosylation pattern of the middle ear mucosa (MEM) of guinea pigs, an approved model for middle ear research, was characterized with the purpose to identify bioadhesive ligands which might prolong the contact time of drug delivery systems with the middle ear mucosa (MEM). To assess the utility of five fluorescein labeled plant lectins with different carbohydrate specificities as bioadhesive ligands, viable MEM specimens were incubated at 4 °C and the lectin binding capacities were calculated from the MEM-associated relative fluorescence intensities. Among all lectins under investigation, fluorescein-labeled wheat germ agglutinin (F-WGA) emerged as the highest bioadhesive lectin. In general, the accessibility of carbohydrate moieties of the MEM followed the order: sialic acid and *N*-acetyl-d-glucosamine (WGA) >> mannose and galactosamine (*Lensculinaris* agglutinin) > *N*-acetyl-d-glucosamine (*Solanumtuberosum* agglutinin) > fucose (*Ulexeuropaeus* isoagglutinin I) >> terminal mannose α-(1,3)-mannose (*Galanthusnivalis* agglutinin). Competitive inhibition studies with the corresponding carbohydrate revealed that F-WGA-binding was inhibited up to 90% confirming specificity of the F-WGA–MEM interaction. The cilia of the MEM were identified as F-WGA binding sites by fluorescence imaging as well as a z-stack of overlays of transmission, F-WGA- and nuclei-stained images of the MEM. Additionally, co-localisation experiments revealed that F-WGA bound to acidic mucopolysaccharides of the MEM. All in all, lectin-mediated bioadhesion to the MEM is proposed as a new concept for drug delivery to prolong the residence time of the drug in the tympanic cavity especially for successful therapy for difficult-to-treat diseases such as otitis media.

## Graphical abstract

**Figure:**
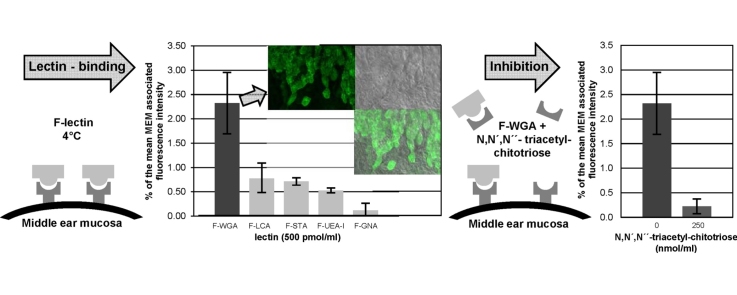

## 1 Introduction

Otitis media (OM) is one of the most common inflammatory diseases of children and therefore the third most common reason for antibiotic therapy in pediatrics (Holstiege et al., 2013). Although specific antibiotics are administered, the overall clinical effectiveness is limited due to low penetration of the drug to the middle ear mucosa (MEM) (Coates et al., 2008), inaccessibility of the bacteria within the grown biofilm (Post et al., 2004) as well as low symptomatic amendment within the first 24 h (Glasziou et al., 2004; Rovers et al., 2006). To increase the therapeutic outcome and to prolong the contact time of the drug with the infected tissue drug loaded formulations such as thermosensitive hydrogels (Lee et al., 2004; Li et al., 2014; Honeder et al., 2014), ototopical drops (Kutz et al., 2013), implants (Goycoolea et al., 1992; Goycoolea and Muchow, 1994; Nether et al., 2004), micropumps (Lehner et al., 1997), intranasal drug delivery systems (Chandrasekhar and Mautone, 2004), coated middle ear prostheses (Lensing et al., 2013; Ehlert et al., 2013; Hesse et al., 2013), and pellets (Daniel et al., 2012) were developed. Although there are many different therapeutic approaches, a local intratympanic therapy seems to be most beneficial for the treatment of OM as a decrease in side effects provoked by systemic therapy as well as an increase in compliance of the young patients will be expected. Nevertheless, the local intratympanic therapy is limited by unfavorable anatomical conditions. The nasopharynx is connected with the tympanic cavity that can lead to rapid drainage of intratympanally administered solutions and suspensions. To avoid Eustachian drainage and to concurrently prolong the contact time of the drug we propose bioadhesive carrier systems interacting with the MEM. As the middle ear is lined with a modified respiratory epithelial layer (Hentzer, 1984) and comprises, among others, ciliated, secretory as well as goblet cells (Lim and Shimada, 1972), the carbohydrates of the glycocalyx at the membrane of these cells might be exploited as bioadhesive sites for glycotargeted delivery. Carbohydrate-binding proteins such as plant lectins interacting with certain sugar residues on the cell surface can function as a ligand. This bioadhesion concept of lectin-mediated targeting has been already reported for overcoming several biological barriers (Bies et al., 2004; Wirth et al., 2002), such as the intestinal epithelium (Gabor et al., 1998), the urothelium (Plattner et al., 2008; Neutsch et al., 2013), the blood–brain barrier (Plattner et al., 2010), and the lymphoid tissue (Diesner et al., 2012).

As a first step toward putting this concept into practice the glycosylation pattern of the MEM has to be elucidated, which is not reported until now to the best of our knowledge. To identify accessible carbohydrate moieties and vice versa appropriate bioadhesive ligands, the interaction of MEM isolated from guinea pigs with a panel of fluorescent labeled lectins with different carbohydrate specificities was investigated: the wheat germ agglutinin (WGA) from *Triticum vulgare* binding to *N*-acetyl-d-glucosamine and sialic acid (Goldstein and Poretz, 1986), the lectin from furze seeds (*Ulex europaeus* isoagglutinin I, UEA-I) which interacts with α-l-fucose-containing carbohydrates (Gürtler, 1978), the α-1,3-mannose-specific *Galanthus nivalis* agglutinin (GNA) (Van Damme et al., 1987), the *Solanum tuberosum* lectin (STA) from potato tubers binding to *N*-acetyl-d-glucosamine (Allen and Neuberger, 1973), and the lentil lectin from *Lens culinaris* (LCA) recognizing galactosaminyl-/mannosyl-residues (Flika et al., 1978). Ongoing from cytoadhesion experiments at 4 °C and cytoinvasion assays at 37 °C, the specificity of interaction will be described. Additionally, co-localisation of lectin-interacting carbohydrates and acidic mucopolysaccharides was applied to identify the lectin binding sites at the MEM.

All in all, this study is aimed to roughly characterize the carbohydrate pattern of the MEM and to identify ligands for glycotargeting as a basis for the development of bioadhesive antibiotic formulations.

## 2 Materials and methods

### 2.1 Materials

The fluorescein-labeled lectins from *T. vulgare* (WGA; wheat germ agglutinin, molar ratio fluorescein/protein (F/P) = 4.5), *S. tuberosum* (STA; F/P = 3.0), *U. europaeus* (UEA-I, isoagglutinin I; F/P = 2.9), *G. nivalis* (GNA; F/P = 5.5), and *Lens culinaris* (LCA; F/P = 3.4) were purchased from Vector Laboratories (Burlingame, CA, USA). Hoechst 33342 trihydrochloride trihydrate was obtained from Invitrogen (Vienna, Austria). Alcian blue and *N*,*N*′,*N*′′-triacetyl-chitotriose were from Sigma–Aldrich (Vienna, Austria). Fluorescein-labeled α-lactalbumin was acquired from Molecular Probes (Eugene, Oregon, USA). All other chemicals were bought from Sigma–Aldrich and were of analytical grade.

### 2.2 Lectin-binding capacity of the MEM

Immediately after sacrificing the guinea pig the bullas were dissected and opened carefully. After rinsing the MEM with 0.9% NaCl the bullas were mounted with the auditory canal upside and the mucosa was incubated with 500 μl solution of fluorescein-labeled lectins (500 pmol/ml 0.9% NaCl) for 30 min at 4 °C or 37 °C. Unbound lectin was removed by washing the cell layer 5 times with 500 μl saline and the nuclei were stained by incubation with 500 μl solution of Hoechst 33342 (0.1 mg/ml 0.9% NaCl) for 10 min at 37 °C. The specimen was washed again thoroughly and the staining pattern of the MEM was fixed by incubation in ice-cold MeOH at −20 °C for 20 min. After rehydration in 0.9% NaCl at room temperature for another 20 min, the MEM was carefully removed from the bulla and mounted on a slide in FluorSave™ for visualization and quantification. To allow comparability of the data, different issues were considered for calculation: (i) since the degree of fluorescein-substitution differs between the lectins, the MFI of each lectin was related to an apparent conjugation number of 1 mol fluorescein per mol lectin according to the fluorescein/protein ratio. (ii) As the size of the specimens is different and sometimes parts of the collected MEM were overlapping, the highest MFI of squares with stained nuclei was set 100% and only squares were considered with a MFI higher than the autofluorescence of the cells. (iii) Only the MFI of cell-associated lectins of nuclei positive squares was considered, related to the MFI of the stained nuclei in these squares, and expressed as a percentage.

As a control to estimate nonspecific binding, samples prepared as described above were treated with a solution of F-lactalbumin instead of the lectins.

### 2.3 Specificity of lectin-binding

To investigate the specificity of the F-WGA–cell interaction competitive inhibition experiments using the complementary carbohydrate *N*,*N*′,*N*′′-triacetyl-chitotriose were performed. After washing the bulla with 0.9% NaCl the MEM was incubated with a freshly prepared mixture of 250 μl solution of the complementary carbohydrate (0–500 nmol/ml) and 250 μl solution of F-WGA (500 pmol/ml) for 30 min at 4 °C. After removal of non-bound lectin and soluble carbohydrate–lectin complexes by thorough washings and preparation of the MEM, the cell-bound fluorescence was determined as described below.

### 2.4 Lectin-uptake by the MEM

In order to find out, whether the MEM-bound lectin is taken up into the cells, a pulse-chase protocol was performed: the MEM was incubated with 500 μl solution of fluorescein-labeled lectins (500 pmol/ml 0.9% NaCl) for 30 min at 4 °C followed by removal of unbound lectin by washing 5 times with saline. The cell-bound lectins were allowed to be internalized during the chase period at 37 °C for another 60 min. Subsequently, the mean MEM-associated fluorescence intensity (MFI) was determined as described below.

### 2.5 Staining of acidic components of the mucosa

After fixing the ear with the auditory canal in an upright position, the bulla was filled with 500 μl 3% acetic acid and incubated for 3 min. This solution was replaced by 500 μl alcian blue solution (10 mg/ml in 3% acetic acid) and removed after 30 min incubation at room temperature (Sheehan and Hrapchak, 1980; Bancroft and Stevens, 1982). The MEM was washed 5 times with aqueous 0.9% NaCl solution and after staining with F-WGA as described above the lectin binding capacity was visualized by microscopy.

### 2.6 Semi-quantitative determination of fluorescence intensity

The relative cell-layer-associated fluorescence intensity of the fluorescein-labeled lectins and the Hoechst 33342 stained nuclei was determined by reading the fluorescence (TECAN, Infinite M200, Austria) at an excitation/emission wavelength of 485/525 nm and 365/450 nm, respectively. The slides were fixed in a frame and the laser was adjusted to read one 3 × 3 mm square after another throughout the whole area of the fixed slide. Cell-layers incubated with buffer served as a control for autofluorescence of cells and slides.

To guarantee comparability of the results and to consider the influence of uneven and sometimes folded surfaces on the quantum yield, the MFI of the nuclei stained with Hoechst 33342 was chosen as a measure for the amount of tissue in each sample. The fluorescein-readouts were related to this area and expressed as a percentage.

### 2.7 Microscopy

To visualize adhesion of the different lectins to the MEM, fluorescence images of the prepared slides were acquired at 20× or 63× magnification with a Zeiss Axio Observer.Z1 microscopy system equipped with the LED illumination system “Colybri” (Zeiss, Göttingen, Germany). For comparability of the images, the excitation exposure time of each lectin was related to an apparent conjugation number of 1 mol fluorescein per mol of lectin by considering the F/P-ratio.

Histological images were obtained using a Nikon Eclipse 50i microscope equipped with an EXFO X-Cite 120 fluorescence illumination system (Nikon, Germany). The images of the FITC-labeled lectins at an excitation/emission wavelength of 465–495/515–555 nm as well as the transmitted light images were acquired at 40× magnification and processed using Lucia G v5.0 software for evaluation.

### 2.8 Statistics

The integrated analysis tools of Microsoft Excel^®^ were used to carry out statistical analyses. The hypothesis test was made by comparing two means from independent samples, among two data sets (*t*-test). Values of *p* < 0.05 were considered as significant. All experiments were performed at least three times.

## 3 Results

To guarantee that the upcoming experiments are performed with viable MEM cells different preparation techniques were tested in preliminary assays using propidium iodide staining of nuclei as a selection criterion (data not shown). In general, incubation in presence of PBS resulted in immediate loss of viability up to 80%. Additionally, detachment of the MEM from the bone matrix followed by incubation with ligands yielded non-utilizable specimens due to folding of the tissue and uneven staining. Only incubation of the bone attached mucosa in presence of saline at 4 °C or 37 °C and subsequent isolation of the MEM just before analysis yielded reliable results with more than 90% viable cells.

### 3.1 Lectin-binding capacity

Preliminary studies were aimed at selection of appropriate concentration ranges for the lectin binding studies. Whereas F-WGA seemed to over-saturate the MEM-specimens at concentrations higher than 1000 pmol/ml, 500 pmol/ml were still sufficient to saturate potential binding sites. Thus this concentration was applied to investigate the glycosylation pattern of the MEM from guinea pigs by use of five fluorescein-labeled lectins with different carbohydrate specificities.

**Fig. 1:**
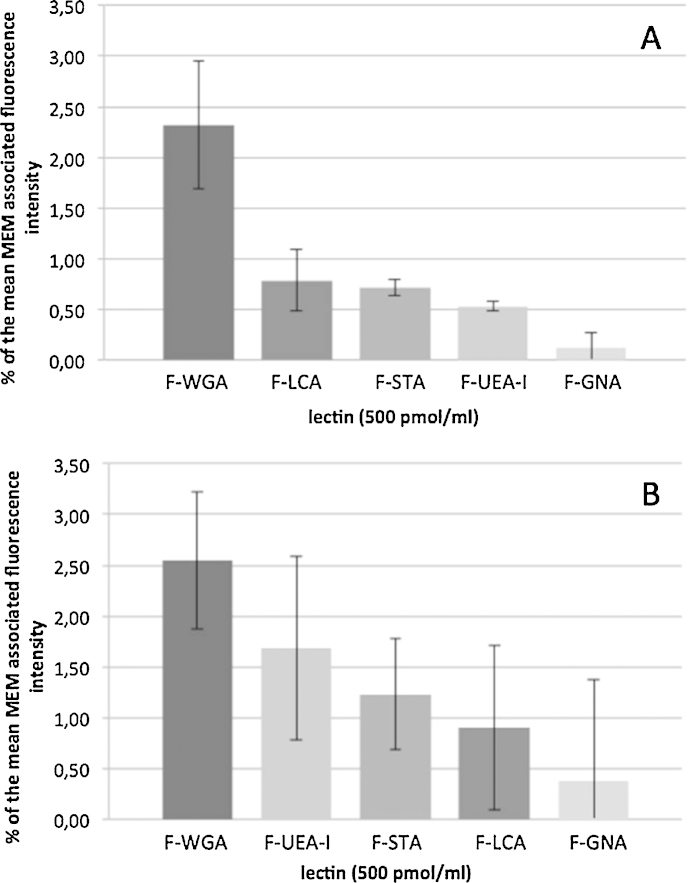
Association of lectins with the MEM at 4 °C (A) or 37 °C (B). For comparability, the cell-associated fluorescein intensity was related to an apparent F/P-ratio of 1 as well as to the fluorescence intensity of stained nuclei (mean ± SD, *n* = 3).

Upon incubation at 4 °C WGA exhibited the highest binding rate with 2.32 ± 0.6% MFI and F-GNA the lowest one close to the autofluorescence of the MEM (Fig. 1A). The intensity of the other lectins was similar amounting to about 0.5% MFI, so that the MEM-associated fluorescence intensities followed the order: WGA >> LCA > STA > UEA-I >> GNA. Besides, the interaction of WGA and all other lectins as well as that between GNL and UEA-I, STA as well as LCA was significantly different (*p* < 0.05). At 37 °C the highest MEM-association capacity was again observed in case of WGA amounting to 2.55 ± 0.7% MFI (Fig. 1B). Nevertheless, this interaction was only significantly different to that of GNA. At the mean, some places changed in the ranking in comparison to 4 °C resulting in the following order: WGA > UEA-I > STA > LCA > GNA.

### 3.2 Microscopical visualization of the binding pattern

**Fig. 2:**
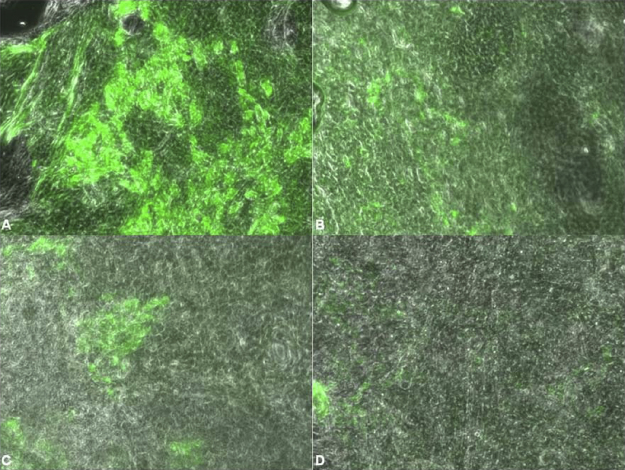
Microscopical visualization and comparison of the lectin binding pattern and intensity of F-WGA (A), F-LCA (B), F-STA (C) and F-UEA-I (D) after incubation at 4 °C by overlaying fluorescence and DIC images. For comparability, the exposure time was correlated to the F/P-ratio of the lectins. Images were acquired at 20× magnification.

The microscopic visualization of the lectin-MEM interaction confirmed the results of the quantitative assay at both temperatures as the MFI of the images decreased according to the ranking described above (Fig. 2). Interestingly, differences in the staining pattern were observed: the *N*-acetyl-d-glucosamine interacting lectins WGA and STA yielded colored clusters but only WGA showed additional less intense as well as diffuse staining. LCA was rather evenly distributed throughout the MEM-surface and UEA-I-binding was lowest but still visible. In contrast, the binding of GNA could not be observed microscopically.

**Fig. 3:**
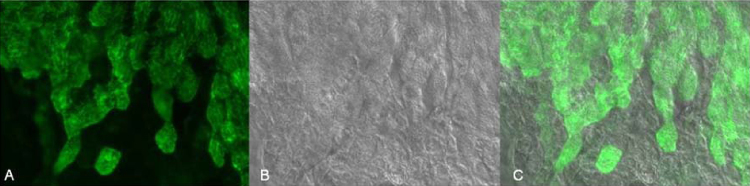
Identification of cilia as a binding site for WGA by fluorescence imaging (A) DIC-imaging (B) and overlay of both (C). Images were acquired at 63× magnification.

**Fig. 4:**
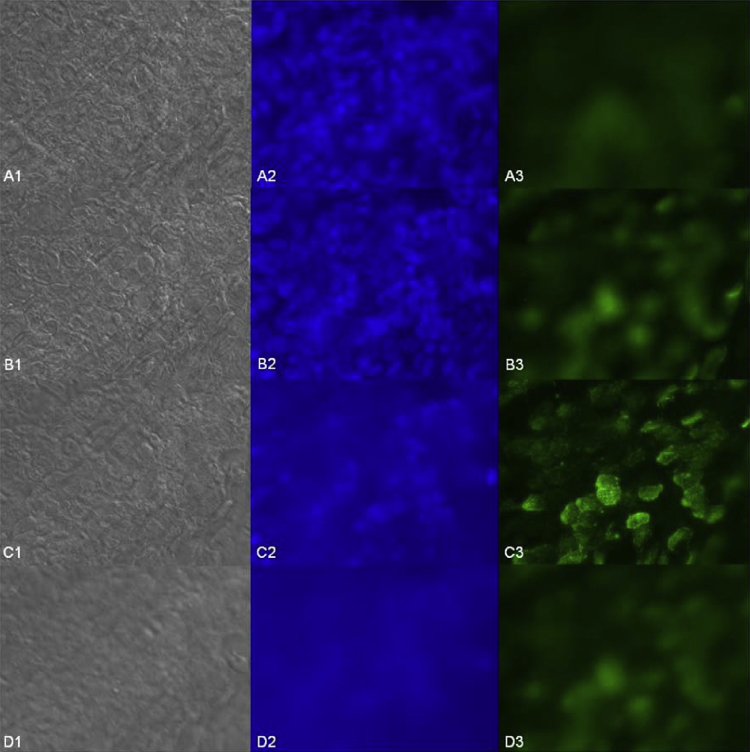
Z-stack of DIC (1), nuclei (2, stained blue) and F-WGA (3, green) at different levels of the MEM: 0 μm (A), 6 μm (B), 12 μm (C) and 18 μm (D). Images were acquired with 63× magnification. (For interpretation of the references to color in this figure legend, the reader is referred to the web version of this article.)

**Fig. 5:**
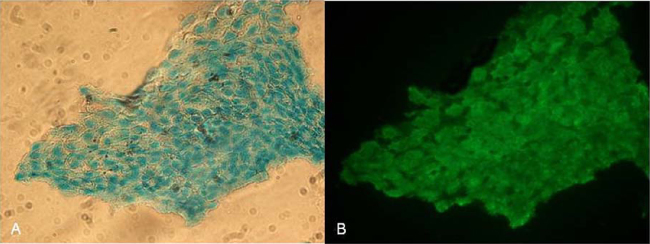
Co-staining of the acidic mucopolysaccharides with alcian blue (A) and sialyl- as well as *N*-acetyl-glucosaminyl residues with F-WGA (B) of the MEM. Images were acquired at 40× magnification.

As WGA proved to highly interact with the MEM, this interaction was elucidated in more detail. The overlay of differential interference contrast (DIC) images and fluorescence images revealed that the WGA-binding pattern coincides with the ciliated cell surface (Fig. 3). Although the exposure time was extended to 3 s binding of F-LCA and F-GNA to ciliated areas was not detectable. To get some further evidence for WGA-binding to the cilia, a z-stack of images was collected. At the level of the nuclei (Fig. 4A) no fluorescence of bound WGA was observed. Moving the focus plane 6 μm higher toward the apical face (Fig. 4B), the nuclei were still visible and a rather weak scattered diffuse fluorescence of WGA was detectable. Analyzing the image acquired another 6 μm higher revealed diffuse blue fluorescence but a sharp F-WGA staining of finger-like structures of the MEM (Fig. 4C), which disappeared again by further rising the focus plane (Fig. 4D). This confirms that WGA predominantly interacts with protruding elements of the MEM such as the cilia. Additionally, as displayed in Fig. 5, co-localization of acidic glycans and the binding site of WGA by co-staining with alcian blue and F-WGA revealed a punctuate pattern on the MEM indicative for acidic polysaccharides as lectin binding sites on the cilia of the MEM.

### 3.3 Specificity of the lectin-binding

Contribution of non-specific protein–protein interactions to the lectin–MEM interaction was investigated by binding studies with F-lactalbumin (Permyakov, 2005). Even in presence of high amounts of lactalbumin up to 2000 pmol/ml, no interaction could be detected. Similarly, the double amount of fluorescein as contained in the highest concentration of F-WGA applied contributed only to 0.02% of MFI.

**Fig. 6:**
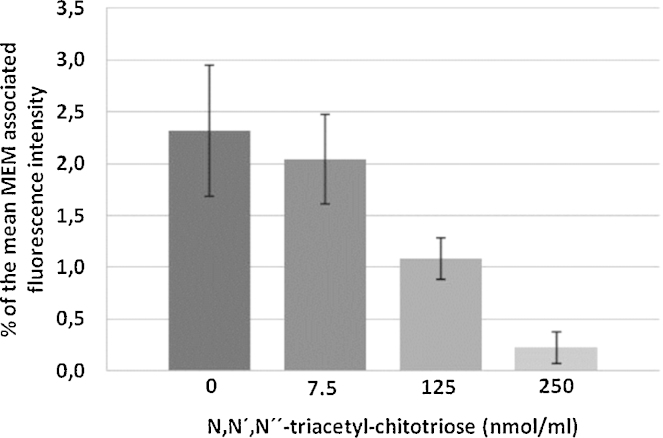
Competitive inhibition of WGA-binding to the MEM by addition of increasing amounts of the complementary carbohydrate.

As only WGA considerably interacted with the mucosa, the specificity of the interaction was elucidated by competitive inhibition of lectin binding sites at the mucosa by addition of the best fitting complementary carbohydrate *N*,*N*′,*N*′′-triacetyl-chitotriose. The MFI of MEM-bound WGA decreased with increasing amounts of the corresponding carbohydrate up to 90% (Fig. 6). The values were significantly different (*p* < 0.05) and confirm high specificity of the WGA–MEM interaction since the adhesion was inhibited by a defined molecule.

### 3.4 Uptake of WGA into MEM-cells

According to the observation that the mean MFI of MEM-bound WGA was slightly, but not significantly, higher at 37 °C than at 4°C the lectin might be bound and taken up into the cell. For that purpose, first, the lectin was bound to the cell-membrane at 4 °C. At this temperature the fluidity of the cell membrane and the metabolism is reduced and energy consuming mechanisms like active transport are repressed. After removal of non-bound lectin, the cells were incubated in a second step at 37 °C to allow for energy dependent uptake processes. Due to shielding of the fluorescence of internalized ligand, the MFI is expected to decrease. Although the MFI was 2.32 ± 0.6% after incubation at 4 °C and decreased to 1.71 ± 0.6% after incubation at 37 °C for 60 min, again the differences were not significant (data not shown). Thus, internalization of membrane-associated lectin could not be confirmed.

## 4 Discussion

In an effort to identify bioadhesive or even cytoinvasive ligands for improved drug delivery in the middle ear, the glycosylation pattern of the MEM from guinea pigs, an approved model for research of OM (Zak and Sande, 1999), was systematically characterized via detailed analysis of the binding capacities of selected fluorescein-labeled lectins with different carbohydrate binding specificities.

For quantitation of the lectin-binding rate and comparability of results, the fluorescein density of the lectins as well as the fluorescein intensity of stained nuclei as a measure for the tissue were considered for calculation. According to the results, the glycocalyx of the MEM contains highest numbers of accessible sialic acid and *N*-acetyl-d-glucosamine moieties and minor amounts of fucosyl- or mannosyl-residues. In case of 37 °C, however, the glycosylation pattern changed following the order: sialic acid and *N*-acetyl-d-glucosamine > fucose > *N*-acetyl-d-glucosamine > mannose and galactosamine > terminal mannose α-(1,3)-mannose. The different glycosylation pattern at the two temperatures might be due to temperature-dependent viscosity of the mucus (Snary et al., 1973) which seems to influence the accessibility of the carbohydrate residues. At body temperature the MEM contains about 3.17-fold higher amounts of fucose and terminal mannose α-(1,3)-mannose as well as 1.73-fold more *N*-acetyl-d-glucosamine residues. The amounts of sialic acid and *N*-acetyl-d-glucosamine or mannose and galactosamine moieties were comparable with those at 4 °C increasing only 1.10- and 1.15-fold, respectively. Since both, WGA and STA interact with *N*-acetyl-d-glucosamine, but WGA additionally with sialic acid, the 3.27-fold higher binding rate of WGA is indicative for presence of high amounts of sialyl-residues. This observation was additionally confirmed microscopically by co-localisation of WGA binding sites and acidic mucopolysaccharides.

For drug delivery to the middle ear not only the binding rate but also the specificity of the interaction is another important parameter. According to competitive inhibition studies WGA specifically interacted with the MEM as indicated by up to 90% inhibition. Additionally, non-specific protein–protein interactions negligibly contribute to lectin–MEM binding. Out of these reasons WGA is an interesting ligand for a lectin-mediated drug delivery to the middle ear. Although the MFI of WGA decreases by 25% only at the mean by increasing the temperature from 4 °C to 37 °C, cytoinvasion of the bioadhesive ligand could not be confirmed. It should be considered, however, that in case of OM the drug should preferably interact after local administration in the tympanic cavity with the cause of disease, the bacteria. Nevertheless, uptake into the MEM-cells might be beneficial to combat also bacteria hidden in the cytoplasm.

The quantitation of lectin binding-efficiency is confirmed qualitatively by fluorescence microscopy revealing the sialyl- and *N*-acetyl-d-glucosamine specific WGA as the most efficient ligand. According to the literature, the composition of the glycocalyx is altered during OM due to upregulation of mucin genes (Ohashi et al., 1989). Since mucin genes encode among others for sialic acid (Lin et al., 2001), the number of potential WGA-binding sites might increase even in case of inflammation and improve the residence time in the tympanic cavity and the efficiency of WGA-grafted formulations.

Histologically, the MEM consists of epithelial-, secretory- and goblet cells, whereas the luminal intratympanic face additionally contains ciliated areas without nuclei (Hentzer, 1984). Applying double staining techniques, those cilia of the MEM surface are identified as a binding site for WGA. This aspect might be beneficial for lectin-mediated drug delivery since mucociliary clearance might decrease the efficacy of non-adhesive formulations. However, according to the literature the ciliary motion is diminished (Ogasawara et al., 2007) during inflammation and might not influence the lectin–MEM interaction.

Another aspect to be considered for use of WGA-functionalized carrier systems for OM-therapy is the formation of a biofilm consisting of bacteria, which overlays the MEM (Post et al., 2004). Consequently, the biofilm can limit accessibility of the MEM and block the docking sites for WGA so that the carrier cannot interact. However, former studies demonstrate that the biofilm is stained with WGA (Thornton et al., 2013). Thus, even the biofilm might be an additional potential bioadhesive site for WGA-grafted drug delivery systems for therapy of OM.

## 5 Conclusion

All in all, the screening of the glycosylation pattern of the MEM by use of lectins with different carbohydrate specificity revealed that sialic acid and *N*-acetyl-d-glucosamine are the most abundant and accessible binding sites of the MEM. Consequently, among the lectins under investigation, WGA emerged as the most promising ligand for drug carrier systems interacting with the cilia of the MEM. Thus, the concept of lectin-mediated bioadhesive drug delivery is proposed as a platform for local intratympanic therapy, which offers prolonged residence time, shortened diffusional pathways and increased concentration gradient that altogether should result in improved efficacy of drugs.
